# Compliance of systematic reviews in ophthalmology with the PRISMA statement

**DOI:** 10.1186/s12874-017-0450-1

**Published:** 2017-12-28

**Authors:** Seon-Young Lee, Harkiran Sagoo, Reem Farwana, Katharine Whitehurst, Alex Fowler, Riaz Agha

**Affiliations:** 1 Nottingham University Hospital, Trent NHS Foundation Trust, Nottingham, UK; 2 Guy’s King’s and St. Thomas’ School of Medical Education, London, UK; 3 University of Birmingham, Birmingham, UK; 4 University College London, London, UK; 5 Guy’s and St. Thomas’ NHS Foundation Trust, London, UK; 6 Guy’s and St. Thomas’ NHS Foundation Trust and Balliol College, University of Oxford, Oxford, UK

**Keywords:** Systematic reviews, Meta-analysis, Research methodology, Reporting quality, PRISMA, Ophthalmology

## Abstract

**Background:**

Systematic reviews and meta-analyses are becoming increasingly important methods to summarize published research. Studies of ophthalmology may present additional challenges because of their potentially complex study designs. The aim of this study was to evaluate the reporting quality of systematic reviews and meta-analyses on topics in ophthalmology to determine compliance with the PRISMA guidelines. We assessed articles published between 2010 and 2015 in the five major relevant journals with the highest impact factors.

**Methods:**

The MEDLINE and EMBASE databases were searched to identify systematic reviews published between January 2010 and December 2015 in the following 5 major ophthalmology journals: Progress in Retinal and Eye Research, Ophthalmology, Archives of Ophthalmology, American Journal of Ophthalmology, and Survey of Ophthalmology. The screening, identification, and scoring of articles were independently performed by two teams, and the results were submitted to statistical analysis to determine medians, ranges, and 95% CIs.

**Results:**

A total of 115 articles were included. The median compliance was 15 out of 27 items (56%), the range was 5–26 (26–96%), and the inter-quartile range was 10 (37%). Compliance was highest in items related to the ‘description of rationale’ (item 3, 100%) and sequentially lower in ‘the general interpretation of results’ (item 26, 96%) and ‘the inclusion of a structured summary in the abstract’ (item 2, 90%). Compliance was poorest in the items ‘indication of review protocol and registration’ (item 5, 9%), ‘specification of risk of biases that may affect the cumulative evidence’ (item 15, 24%), and ‘description of clear objectives in the introduction’ (item 4, 26%).

**Conclusion:**

The reporting quality of systematic reviews and meta-analyses in ophthalmology should be significantly improved. While we recommend the use of the PRISMA criteria as a guideline before journal submission, additional research aimed at identifying potential barriers to compliance may be required to improve compliance with PRISMA guidelines.

**Electronic supplementary material:**

The online version of this article (10.1186/s12874-017-0450-1) contains supplementary material, which is available to authorized users.

## Background

Systematic reviews and meta-analyses are key study designs that are used to summarize published research findings that are replacing traditional reviews and expert commentaries. In these studies, the authors collect and critically assess all evidence that fits pre-specified criteria with the aim of answering a specific research question. They are therefore able to limit biases by including a wider range of populations than can be included in a single centre study. Moreover, they may quantify the heterogeneity in the available data and allow the results of multiple studies to be pooled into a meta-analysis, thereby increasing statistical power. In many cases, the goal is to identify the most appropriate treatment for a particular clinical paradigm [1–3]. Reliance on systematic reviews and meta-analysis by health care practitioners and policy makers, including those involved in ophthalmology research, in which approximately 1000 systematic review publications have been published, is increasing [4].

The PRISMA (Preferred Reporting Items for Systematic Reviews and Meta-Analyses) statement was published in 2009 and consists of a 27-item checklist that covers each section of an article and includes a flow diagram. Its purpose is to help authors improve the quality of reporting in systematic reviews and meta-analyses [5]. Similar studies have been reported in different fields, including orthorhinolaryngology [6], orthodontics [7], radiology [8], gastroenterology and hepatology [9], and orthopaedics [10]. In a previous study, we also assessed the compliance of articles related to plastic surgery to the PRISMA statement. Our results showed that the reporting quality in plastic surgery was suboptimal [11].

Regardless of the field of research, poorly reported systematic reviews can be misleading and potentially dangerous in clinical practice. Studies in ophthalmology may present additional challenges to both researchers and readers because of the need for complexity in study designs. For example, there are two potential data points in each patient (i.e., two eyes), and accounting for this fact may require alternative designs and methods of analysis. Hence, the results and conclusions of such studies may be confusing if they do not provide an appropriate level of explanation [12]. Systematic reviews play a role in enabling the accurate appraisal of the literature because they allow large pools of data to be simultaneously interpreted, which can resolve some of the complexity in this field. It is also important for the authors of these studies to provide complete, clear and transparent information by using good reporting methods [5, 13]. The PRISMA statement is an objective index that can be used to determine whether systematic reviews are adequately performing this role.

The aim of this study was to determine the reporting quality of systematic reviews and meta-analyses in ophthalmology according to the PRISMA statement. We evaluated articles published between 2010 and 2015 from five major journals with the highest impact factor.

## Methods

This study is registered at Research Registry (unique identifying number: researchregistry952; http://www.researchregistry.com). Institutional review board approval was not required because the study did not involve human participants.

### Search methods

The MEDLINE and EMBASE databases were searched to identify systematic reviews published between January 1, 2010 and December 31, 2015 in the five major ophthalmology journals with the highest impact factor (IF) according to the Thomson Reuter Impact factor 2014 [14]: The following journals were included: Progress in Retinal and Eye Research (IF 8.733), Ophthalmology (IF 6.135), Archives of Ophthalmology (IF 4.399), American Journal of Ophthalmology (IF 3.871), and Survey of Ophthalmology (IF 3.507), all of which represent a significant output for the field. The search strategies used in each database are described in Additional file 1.

The articles were manually and independently screened by three researchers, (H.S., K.W. and R.F.) who assessed their titles and abstracts. The following inclusion criteria were used: systematic reviews and/or meta-analyses published between January 2010 and December 2015. Any non-systematic reviews were excluded. Definition to identify systematic reviews has been adopted from PRISMA and the Cochrane Collaboration [15]. Examples of excluded works included narrative reviews, expert reviews and randomized controlled trials. Any disagreements were forwarded to another member of the team (S.Y.L.) for a final decision.

### Scoring

Articles were scored against the 27 checklist items described in the PRISMA 2009 statement [16] by two independent researchers (H.S., and R.F.). Each item was weighted equally, and the same number of denominators was applied to each article. A score was computed for each article. Items received a mark only when the article fulfilled the criteria described within each item of the PRISMA statement. If the article did not explicitly mention the terms specified in the guidelines, efforts made to report the requirements per the guidelines were considered. For example, if the article did not specifically use the term ‘bias’, if an effort was made to assess the risk of bias, the relevant items were still given marks. Any description of a justification to not fulfil a criterion was also given a mark. For instance, we scored the article as fulfilled if it justified why meta-analysis was not performed. In cases where the PRISMA item included multiple elements, marks were given when all elements were reported. Discrepancies were initially solved by consensus, and any remaining disagreements were referred to another member of the team (S.Y.L.).

### Statistical analysis

All statistical analyses were performed using Microsoft Excel 2011 (Microsoft Corporation). Descriptive statistics, including the median and range and their 95% Confidence Intervals (CIs), were calculated. The level of compliance with individual items was computed for each article as a percentage. Cohen’s Kappa statistic was used to calculate inter-observer agreement in scoring between two researchers. Cohen’s Kappa statistic measures the magnitude of agreement that goes beyond chance alone [17].

## Results

Among the 5 journals included in this study, only one journal (Ophthalmology) required the PRISMA checklist to be used to report a systematic review or meta-analysis.

**Fig. 1:**
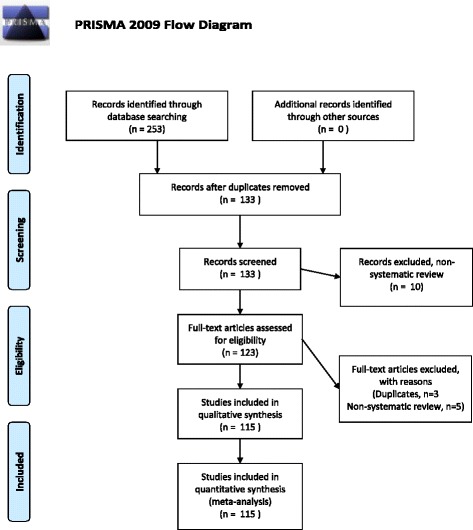
Flow diagram describing process of articles being reviewed and selected

The initial electronic search identified 253 articles. Of these, 115 were selected for data extraction (Fig. 1). Arbitration was required to determine whether to include 8 articles, and disagreement occurred regarding 375 points across the 115 articles. The Cohen’s Kappa statistic was 0.65, which indicates a good level of agreement between scorers.

**Fig. 2:**
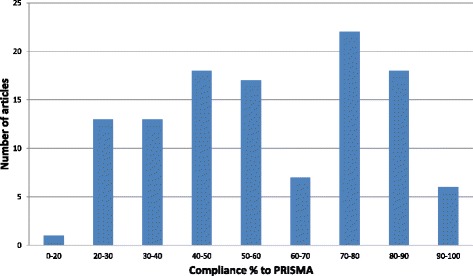
The number of articles according to their compliance range with the PRISMA criteria

The median compliance across the 115 included articles was 15 out of 27 items (56%) and had a range of 5–26 (19–96%) and an inter-quartile range of 10 (37%). The compliance of the articles with each PRISMA item showed wide variance (Table 1). More than half of the articles achieved more than 50% compliance with the PRISMA checklist. However, approximately 10% of the articles failed to achieve compliance higher than 30%, and no single article met all 27 criteria (Fig. 2).

**Table 1:** Compliance with PRISMA checklist items (Table adapted from the PRISMA checklist, Moher et al.) [15]

Section/Topic	No.	Brief description of the item	Compliance
TITLE			
Title	1	Identification of the report	62 (54%)
ABSTRACT			
Structured summary	2	Provide a structured summary	103 (90%)
INTRODUCTION			
Rationale	3	Background rationale	115 (100%)
Objectives	4	Description of PICOS (Participants, Interventions, Comparisons, and Study design)	30 (26%)
METHODS			
Protocol and registration	5	Indication of review protocol & registration information	10 (9%)
Eligibility criteria	6	Specification of study and review characteristics used as eligibility criteria	91 (79%)
Information sources	7	Describe all information sources and date last searched.	97 (84%)
Search	8	Present repeatable full electronic search strategy for at least one database	55 (48%)
Study selection	9	State the process for selecting studies	86 (75%)
Data collection process	10	Describe the method of data extraction	78 (68%)
Data items	11	Report all variables and any assumptions and simplifications made.	77 (67%)
Risk of bias in individual studies	12	Describe the methods used to assess the risk of bias of individual studies	46 (40%)
Summary measures	13	State the principal summary measures	71 (62%)
Synthesis of results	14	Describe the methods used to handle and analyse the data	53 (46%)
Risk of bias across studies	15	Specify any assessment of risk of bias that may affect the cumulative evidence	27 (24%)
Additional analyses	16	Describe methods of additional analyses	38 (33%)
RESULTS			
Study selection	17	Give numbers of studies at each stage of the study.	73 (64%)
Study characteristics	18	For each study, present characteristics for which data were extracted	96 (84%)
Risk of bias within studies	19	Present data on risk of bias for each study	42 (37%)
Results of individual studies	20	Report the summary of each data intervention group and estimates of confidence intervals	65 (57%)
Synthesis of results	21	Present the results of each meta-analysis	61 (53%)
Risk of bias across studies	22	Present the results of any assessment of risk of bias across studies	35 (30%)
Additional analysis	23	Give the results of additional analyses	33 (29%)
DISCUSSION			
Summary of evidence	24	Summarize the main findings, including the strength of the evidence	98 (85%)
Limitations	25	Discuss limitations at the study, outcome & review levels	80 (70%)
Conclusions	26	Provide a general interpretation of the results	110 (96%)
FUNDING			
Funding	27	Describe sources of funding & other support	85 (74%)
OVERALL ADHERENCE: 15 (56%)

Meanwhile, compliance with individual items was variable, with three items achieving a level of compliance equal to or greater than 90%, while compliance was less than or equal to 30% for 5 items. The compliance was highest for items related to ‘the description of the rationale for the study’ (item 3, 100%). This was followed by the ‘general interpretation of results’ (item 26, 96%) and the ‘inclusion of a structured summary in the abstract’ (item 2, 90%). Compliance was poorest for ‘indication of review protocol and registration’ (item 5, 9%), ‘specification of risk of bias that may have affected the cumulative evidence’ (item 15, 24%), and ‘description of clear objectives in the introduction’ (item 4, 26%).

## Discussion

The median compliance was 15 out of 27 (56%), which is relatively low in comparison with most previous studies that investigated the reporting quality of systematic reviews in different specialties [8–11, 18]. Half of the articles (62 articles) achieved a compliance of less than 60% (Fig. 2), and no single article met all 27 criteria of the PRISMA statement. Considering that only one journal required the use of a PRISMA checklist prior to the submission of a manuscript presenting a systematic review or meta-analysis, there is a significant amount of room in which improvement can be made in reporting the quality of systematic reviews in ophthalmology.

Five items with compliance less than or equal to 30% include items related to describing the review protocol and registration, describing the risk of bias, describing the objectives using PICOS, and describing additional analysis (items 5, 15, 4, 23, and 22, respectively). Interestingly, poor compliance with these items has also been noted in other previous studies that examined the reporting quality of systematic reviews in different specialties [6–11, 18]. Regardless of their overall compliance with the PRISMA checklist, previous studies in specialties including orthorhinolaryngology [6], orthodontics [7], and radiology [8] all achieved compliance lower than 30% in items relating to describing the review protocol and registration and the risk of bias. A study of journals in gastroenterology and hepatology [9] showed that there was generally high median compliance with the PRISMA checklist (above 80%) but poor compliance (less than 20%) in describing the protocol and registration. Similar studies that evaluated orthopaedic journals [7], our previous study that assessed the compliance of plastic surgery articles to PRISMA [11], and a study of nursing journals [18] produced overlapping results that showed there was low compliance regarding items related to the protocol and registration, the description of bias, and the description of additional analyses. Moreover, a review of 300 systematic reviews [19] found that just under a quarter (23.1%) of authors reported assessing possible publication bias and that no single article in the study mentioned the review protocol or review registration.

The generally low compliance observed in this study is an indication that there is a lack of awareness regarding the importance of review protocols and registration information among authors. The use of protocol and review registration is important because a protocol allows more accurate comparisons to be made between authors and leaders and supports better transparency during the review process [5, 20]. There are web database registration sites that authors can easily use to obtain information regarding protocol and registration. These include the PROSPERO [20] and Research registry [21] databases, which we recommend. It is also disappointing that most specialties achieve a low level of compliance in items related to the assessment of risk of bias. The attempt to eliminate bias is a very fundamental component of randomized controlled trials, as is the assessment of bias in systematic reviews and meta-analysis [22]. Therefore, the poor compliance with items relating to assessing bias in most specialties is actually surprising. We suspect that this is caused by a lack of awareness regarding essential points of research reporting among authors because we noticed that many of the articles briefly mentioned ‘bias’ but then failed to describe the methods or results of their assessment of the risk of bias.

While improved awareness among stakeholders (i.e., journal reviewers and editors, funders, institutions, and readers) is required, we reiterate our previous call for journals to make the PRISMA checklist mandatory for the electronic submission of systematic reviews [11]. Enforcing these guidelines at the time of journal submission may improve the quality of reporting, and their endorsement has been shown to improve adherence [9]. Unfortunately, it is difficult to comment on this point with regard for the five ophthalmology journals included in this study because only one of them required the PRISMA checklist. However, it was recently demonstrated that journals increased their endorsement of reporting guidelines in their introduction to the authors between 2011 and 2014 (10% vs. 19%) [23]. The number of systematic reviews and meta-analyses published in the field of ophthalmology has dramatically increased over the last decade [24, 25]. While this is a positive indication that there is adequate recognition of the increasing number of original high-quality articles published in this field, it also means that there is increased room for error due to the large amount of data. Increased importance should therefore be placed on accurate and detailed reporting [25]. Clear and transparent information not only allows an adequate interpretation of results but also helps authors to correct errors during earlier stages of the study [5, 13].

Some limits need to be discussed regarding the methods used in our research. Because we focused on journals with high impact factors, our findings do not necessarily reflect adherence to PRISMA for systematic reviews published in journals with low impact factors. Additionally, the articles were electronically searched instead of handsearched, the latter of which would have ensured that we retrieved all articles that met the eligibility criteria. However, it was difficult to obtain access to paper copies of the journals, and using electronic search and screening procedures reduced the time that was consumed during the data search phase of this study. Additionally, we believe that MEDLINE and EMBASE cover a sufficient breath of articles (each contains over 20 million records) [26, 27].

We assigned equal weights to all items in the PRISMA checklist and used the same number of denominators in each article. However, this imposed a limitation on our study in that certain items on the checklist may have more impact than others on the reporting quality of systematic reviews. For example, it may be useful to weigh items related to the description of detailed methods more heavily than those related to the identification of the report as a systematic review in the title. Additionally, certain elements in the PRISMA checklist did not apply in some articles. For example, if the systematic review did not include a meta-analysis, items relating to meta-analyses, such as the presentation of the results of each meta-analysis (item 21), are not applicable to the review. Although we considered the article as fulfilled if it justified why meta-analysis was not performed, most articles failed to state such justification. Furthermore, the scoring of some items could vary according to the publishing style of each journal. For example, item 2 requires authors to provide a structured summary that includes many characteristics, such as the background, objective, data sources, and study eligibility criteria. However, compliance with this item can vary depending on how many words are used and what structure each journal requires the authors to provide. We attempted to be generous with such elements, but it remains possible that some articles may have lost a mark as a result of this type of limitation.

Systematic reviews in ophthalmology involve methodological issues that are specific to eyes and vision search, e.g. unit of analysis; we have not explicitly evaluated reporting of such items in this study. Because PRISMA was designed to cover a wide range of specialties, it has some limitations with regard for covering the specific details required in each specialty. In ophthalmology, PRISMA does not consider presentation of two sets of data, one from each eye (i.e., the right and left eye of each patient), a point that becomes even more complicated in studies wherein data can be obtained for only one eye in some patients [2].

Potential barriers to compliance with the PRISMA checklist may include a lack of awareness or understanding of the PRISMA guidelines, which we suspect is the most common cause of lack-of-compliance [28]. However, further study is required to assess such barriers among key stakeholders in ophthalmology because no research into these barriers to compliance with PRISMA has been published.

## Conclusions

The reporting quality of systematic reviews and meta-analysis in ophthalmology needs to be significantly improved, especially in describing the review protocol and registration, describing the risk of bias, describing the objectives, and describing additional analysis. We strongly suggest that journals require that all PRISMA criteria should be fulfilled before allowing electronic journal submission because we believe that this will eliminate ambiguity in the reporting of studies. Additional research aimed at identifying potential barriers to compliance may be required to improve compliance with the PRISMA guidelines.

## Additional files

Additional file 1: Search strategies. Search strategies used to identify systematic reviews and meta-analysis published from major ophthalmology journals between 2010 and 2015, via Medline and Embase. (PDF 13 kb)

Additional file 2: Dataset of research. Raw data used for analysis and interpretation. (XLSX 48 kb)

## Abbreviations

IF: Impact Factor
PICO: Patient/Population/Problem, Intervention, Comparison, Outcome
PRISMA: the Preferred Reporting Items for Systematic Reviews and Meta-Analyses
