# Supplementary material for: Compliance of systematic reviews in ophthalmology with the PRISMA statement
Source: BMC Med Res Methodol. 2017 Dec 28;17:178. doi: 10.1186/s12874-017-0450-1 (PMC5745614; doi:10.1186/s12874-017-0450-1)
Supplement: Supplementary file 1 — Search strategies. Search strategies used to identify systematic reviews and meta-analysis published from major ophthalmology journals between 2010 and 2015, via Medline and Embase. (PDF 13 kb) [file 12874_2017_450_MOESM1_ESM.pdf]

**Appendix 1. Search strategy for MEDLINE**

Database: Ovid MEDLINE(R) <1946 to June Week 5 2017>

Search Strategy:

--------------------------------------------------------------------------------

| | |
|---|---|
| 1 | Meta-Analysis as Topic/ (16173) |
| 2 | meta analy$.tw. (94606) |
| 3 | metaanaly$.tw. (1630) |
| 4 | Meta-Analysis/ (82414) |
| 5 | (systematic adj (review$1 or overview$1)).tw. (82356) |
| 6 | exp Review Literature as Topic/ (9592) |
| 7 | or/1-6 (175190) |
| 8 | cochrane.ab. (45210) |
| 9 | embase.ab. (46576) |
| 10 | (psychlit or psyclit).ab. (899) |
| 11 | (psychinfo or psycinfo).ab. (12040) |
| 12 | (cinahl or cinhal).ab. (15086) |
| 13 | science citation index.ab. (2394) |
| 14 | bids.ab. (393) |
| 15 | cancerlit.ab. (629) |
| 16 | or/8-15 (72903) |
| 17 | reference list$.ab. (12571) |
| 18 | bibliograph$.ab. (13350) |
| 19 | hand-search$.ab. (4755) |
| 20 | relevant journals.ab. (896) |
| 21 | manual search$.ab. (2946) |
| 22 | or/17-21 (30971) |
| 23 | selection criteria.ab. (23960) |
| 24 | data extraction.ab. (12994) |
| 25 | 23 or 24 (35054) |
| 26 | Review/ (2220914) |
| 27 | 25 and 26 (25241) |
| 28 | Comment/ (659756) |
| 29 | Letter/ (933659) |
| 30 | Editorial/ (409271) |
| 31 | animal/ (6164834) |
| 32 | human/ (17014592) |
| 33 | 31 not (31 and 32) (4403138) |
| 34 | or/28-30,33 (5829957) |
| 35 | 7 or 16 or 22 or 27 (208758) |
| 36 | 35 not 34 (196596) |
| 37 | progress in retinal & eye research.jn. (559) |
| 38 | ophthalmology.jn. (14401) |
| 39 | "archives of ophthalmology".jn. (17359) |
| 40 | "american journal of ophthalmology".jn. (24966) |
| 41 | "survey of ophthalmology".jn. (2588) |
| 42 | 37 or 38 or 39 or 40 or 41 (59873) |
| 43 | limit 42 to yr="2010 - 2015" (6834) |
| 44 | 36 and 43 (131) |

**Appendix 2. Search strategy for EMBASE**

Database: Embase <1996 to 2017 Week 28>
Search Strategy:
--------------------------------------------------------------------------------

| | |
|---|---|
| 1 | exp Meta Analysis/ (126000) |
| 2 | ((meta adj analy$) or metaanalys$).tw. (146415) |
| 3 | (systematic adj (review$1 or overview$1)).tw. (128280) |
| 4 | or/1-3 (254153) |
| 5 | cancerlit.ab. (685) |
| 6 | cochrane.ab. (68344) |
| 7 | embase.ab. (70475) |
| 8 | (psychlit or psyclit).ab. (954) |
| 9 | (psychinfo or psycinfo).ab. (16595) |
| 10 | (cinahl or cinhal).ab. (20805) |
| 11 | science citation index.ab. (2911) |
| 12 | bids.ab. (470) |
| 13 | or/5-12 (109176) |
| 14 | reference lists.ab. (14843) |
| 15 | bibliograph$.ab. (15974) |
| 16 | hand-search$.ab. (6433) |
| 17 | manual search$.ab. (3904) |
| 18 | relevant journals.ab. (1148) |
| 19 | or/14-18 (37844) |
| 20 | data extraction.ab. (17257) |
| 21 | selection criteria.ab. (27013) |
| 22 | 20 or 21 (42559) |
| 23 | review.pt. (1828036) |
| 24 | 22 and 23 (20465) |
| 25 | letter.pt. (686641) |
| 26 | editorial.pt. (450176) |
| 27 | animal/ (778374) |
| 28 | human/ (13197255) |
| 29 | 27 not (27 and 28) (551090) |
| 30 | or/25-26,29 (1680104) |
| 31 | 4 or 13 or 19 or 24 (298788) |
| 32 | 31 not 30 (290619) |
| 33 | "progress in retinal and eye research".jn. (671) |
| 34 | ophthalmology.jn. (10918) |
| 35 | "archives of ophthalmology".jn. (6182) |
| 36 | "american journal of ophthalmology".jn. (9457) |
| 37 | "survey of ophthalmology".jn. (1549) |
| 38 | 33 or 34 or 35 or 36 or 37 (28777) |
| 39 | limit 38 to yr="2010 - 2015" (7133) |
| 40 | 32 and 39 (122) |
